# Mental health literacy in children and adolescents in low- and middle-income countries: a mixed studies systematic review and narrative synthesis

**DOI:** 10.1007/s00787-022-01997-6

**Published:** 2022-05-15

**Authors:** Laoise Renwick, Rebecca Pedley, Isobel Johnson, Vicky Bell, Karina Lovell, Penny Bee, Helen Brooks

**Affiliations:** Division of Nursing, Midwifery and Social Work, Faculty of Medicine, Biology and Health, School of Health Sciences, University of Manchester, Room 6.304 Jean McFarlane Building, Oxford Road, Manchester, M13 9PL UK

**Keywords:** Mental health literacy, Children and adolescent, Stigma, Disorder recognition, Help-seeking, Public health

## Abstract

Mental illnesses are the leading cause of disease burden among children and young people (CYP) globally. Low- and middle-income countries (LMIC) are disproportionately affected. Enhancing mental health literacy (MHL) is one way to combat low levels of help-seeking and effective treatment receipt. We aimed to synthesis evidence about knowledge, beliefs and attitudes of CYP in LMICs about mental illnesses, their treatments and outcomes, evaluating factors that can enhance or impede help-seeking to inform context-specific and developmentally appropriate understandings of MHL. Eight bibliographic databases were searched from inception to July 2020: PsycInfo, EMBASE, Medline (OVID), Scopus, ASSIA (ProQuest), SSCI, SCI (Web of Science) CINAHL PLUS, Social Sciences full text (EBSCO). 58 papers (41 quantitative, 13 qualitative, 4 mixed methods) representing 52 separate studies comprising 36,429 participants with a mean age of 15.3 [10.4–17.4], were appraised and synthesized using narrative synthesis methods. Low levels of recognition and knowledge about mental health problems and illnesses, pervasive levels of stigma and low confidence in professional healthcare services, even when considered a valid treatment option were dominant themes. CYP cited the value of traditional healers and social networks for seeking help. Several important areas were under-researched including the link between specific stigma types and active help-seeking and research is needed to understand more fully the interplay between knowledge, beliefs and attitudes across varied cultural settings. Greater exploration of social networks and the value of collaboration with traditional healers is consistent with promising, yet understudied, areas of community-based MHL interventions combining education and social contact.

**Supplementary Information:**

The online version contains supplementary material available at 10.1007/s00787-022-01997-6.

## Introduction

Mental illnesses are the leading cause of disease burden among children and young people (CYP) globally [1] and low-and middle-income countries (LMIC) are disproportionately affected. LMIC populations are predominantly young [2] and are vulnerable to developing mental disorders [3]. Half of all lifetime mental illnesses begin by adolescence which portends markedly worse outcomes than onset later in life [4, 5]. The Lancet Commission on child health and wellbeing identifies that mental health problems are becoming dominant among CYP and substantial investment in prevention approaches is required [6]. There are considerably lower rates of recognition and treatment of mental illness in LMICs [7]. Evidence reports the gap between those needing treatment and those receiving it is up to 90% [8]. Structural barriers including inadequate funding, lack of resources and trained personnel, sub-optimal infrastructure and poorly integrated health systems all contribute to inaccessible mental health care [9]. In LMICs, fewer people seek professional help compared to in high-income settings and where they do, there are long delays with variable pathways [10].

Enhancing mental health literacy (MHL) is one way to combat excessively high rates of undertreatment of mental illnesses among CYP. However, understanding current knowledge and attitudes towards mental illnesses is an important first step towards developing an evidence base and interventions to improve literacy. MHL refers to the “knowledge and beliefs about mental disorders which aid their recognition, management or prevention” [11] and comprises several, interlinked components that include: (a) the ability to recognise specific disorders or different types of psychological distress; (b) knowledge and beliefs about risk factors and causes; (c) knowledge and beliefs about self-help interventions; (d) knowledge and beliefs about professional help available; (e) factors and attitudes which facilitate recognition and appropriate help-seeking; and (f) knowledge of how to seek mental health information. MHL proponents reason that obtaining adequate knowledge of how and when disorders develop and appraising the need for help increases help-seeking behaviours boosting the chance of receiving appropriate and effective treatment [12, 13]. In LMICs, targeting MHL is a specific recommendation for improving the health and wellbeing of younger populations [14]. Evidence from two separate systematic reviews shows that perceived lack of knowledge about mental health problems is one of the most prominent barriers to intended help-seeking reported from the perspective of young people [15, 16]. Research also shows that negative attitudes about mental illness and treatment deters people from seeking help [17].

To promote conceptual clarity, we view MHL as a theory that contains multiple constructs for the present review [18]. This allows clearer formulation of hypothesised relationships, better articulation and understanding of the interrelationships between constructs and outcomes of interest and the development of testable theories regarding the extent that MHL influences mental health behaviours. For the purposes of this review, we define MHL as knowledge, attitudes and beliefs about mental disorders which aids recognition, management or prevention incorporating recognition of developing disorders and effective help-seeking strategies [12]. Jorm’s theory encapsulates knowledge of professional help-seeking, self-treatment and how to seek information to effectively support individual’s to seek appropriate and effective treatments for themselves and to assist others [12] which may have additional relevance in LMIC settings which are broadly defined as collectivist cultures where families and communities take a prominent role in decision-making about health [19]. Help-seeking has particular salience for enhancing capacity in the demand for services and supporting efforts to contribute to closing the significant treatment gap in LMICs [6]. We have expressly included attitudes and beliefs that promote recognition, incorporating stigma, as there is inconsistent evidence of the interrelationship between mental health knowledge and stigma [20–22]. Current evidence from a meta-analysis demonstrates that stigmatising attitudes towards people with mental illness significantly predicts actively seeking treatment for mental health problems [23] and as such is a significant barrier to accessing adequate treatment.

Existing synthesised evidence of MHL in children and young people in LMICs is lacking. Two reviews examine adolescent help-seeking and the relationship with MHL [15, 16]; however, these are not specific to LMICs and less than 8% of included studies originated in LMICs. A single review helpfully examines the conceptualisation of MHL in adolescent populations [14] but again is largely focused on evidence from high-income settings and of the 91 studies included just 6 were conducted in low-resource settings. To ensure context-specific and developmentally appropriate MHL constructs are available, we aim to synthesise evidence about the knowledge, beliefs and attitudes of CYP in LMICs about mental illnesses, their treatments and outcomes, evaluating factors that can enhance or impede help-seeking.

## Methods

### Eligibility criteria

We sought to identify studies reporting primary evidence regarding MHL; thus, we included research that examined perceptions, views and attitudes about mental illnesses and treatment. Studies exploring disorder recognition, knowledge about causes, help-seeking and self-help including factors influencing these MHL components were eligible for inclusion. Participant groups were included if they were under 18 years of age and where this was not clear, we included studies with population group mean age under 18. LMICs were defined by Organization for Economic Cooperation and Development (OECD) Development Assistance Committee (DAC) between 2018 and 2020.^1^

We included peer reviewed journal articles and dissertations which reported primary data. There were no restrictions on study type or the date the study was completed. Conference paper authors were contacted by email to request published reports of conference proceedings. These were excluded if complete information could not be provided by researchers. All languages were included. Data from non-English papers were screened and extracted in different languages by bilingual researchers affiliated with and supported by the study team. Six non-English papers were reviewed at title and abstract screening (Chinese, Portuguese, Spanish, Serbian). Two were excluded at full-text review (Chinese) which resulted in 4 non-English papers (Portuguese, Serbian, Spanish). Inclusion/exclusion criteria are described in Additional File 1.

### Search strategy

Eight bibliographic databases were searched from inception to July 2020: PsycInfo, EMBASE, Medline (OVID), Scopus, ASSIA (ProQuest), SSCI, SCI (Web of Science) CINAHL PLUS, Social Sciences full text (EBSCO). Test searches were completed between October 2018 and January 2019 iteratively readjusting and refining the search strategy. Initial searches were conducted in January 2019 and updated in July 2020. The search strategy incorporated four key areas comprising respondent views and attitudes, mental health, mental illnesses and emotional well-being, children and young people and originating from LMIC. Searches terms included ‘mental illness’, ‘low- and middle-income countries’, ‘perception’, ‘child’ and ‘adolescent’ using synonyms, truncations, and wildcards. An example search strategy is available in Additional File 2. Forward citation tracking was utilised for included papers from January 2019 until April 2020 in both Web of Science and Scopus [24]. The methods and results are reported consistent with PRISMA (Preferred Reporting Items for Systematic Reviews and Meta-Analyses) guidelines [25]. This review has been registered in PROSPERO (CRD42019122057). A sample search is described in Additional File 2.

### Data extraction and appraisal

Included studies were independently screened with each title and abstract screened by two separate researchers from the study team. Full-text review was subsequently conducted by two independent reviewers within the study team. Conflicts were assessed by two members of the study team who were not involved in the original decision (LR and HB) until consensus was reached. Approximately 95% concordance was reached between reviewers at full-text review.

Data were extracted to an Excel sheet developed purposely and piloted prior to review. Qualitative and quantitative outcome data were extracted simultaneously including year of publication, country, and setting (community, school-based, clinical), study design, primary aim, MHL definition and use of scale, inclusion/exclusion and analysis. Extraction allowed for both deductive coding to existing mental health literacy components and inductive coding to allow for the inclusion of data that fell out with the MHL framework [11]. Quality appraisal comprised the Mixed Methods Appraisal Tool (MMAT) [26] designed to appraise five classes of research in mixed studies systematic reviews. Articles were not excluded based on quality assessment as empirical evidence is lacking to support exclusion based on quality criteria [27, 28]. Scores were expressed as a percentage of possible items divided by affirmative items. Each study was then classified as weak (≤ 50%), moderate–weak (51–65%), moderate–strong (66–79%), or strong (≥ 80%) based on a methodological scoring system [29].

### Data analysis and synthesis

**Fig. 1:**
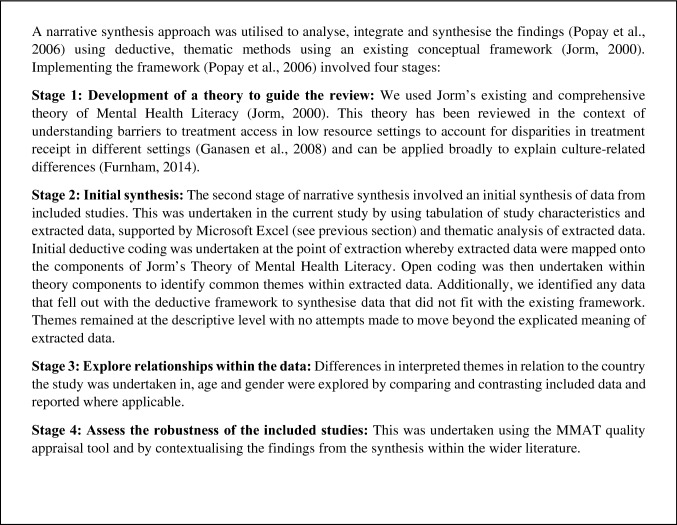
Synthesis process

A narrative synthesis of qualitative and quantitative studies was conducted because of marked methodological and clinical heterogeneity between studies [30]. Using deductive and inductive thematic methods within an existing conceptual framework [11], the synthesis involved four stages [30]. See Fig. 1 for details of the synthesis process. To evaluate the robustness of our synthesis, we examined the findings within the analysis contained in each of our MHL conceptualisations when a) studies with weak quality scores were removed and b) studies with moderate–weak scores were removed. We then assessed the contribution of each individual piece of evidence to the consistency of descriptions within our synthesis to examine whether different pieces of information were compatible with the overall synthesis. Given the volume of evidence in included manuscripts, we presented the dominant themes and higher-order categories in our synthesis.

## Results

### Study characteristics

**Fig. 2:**
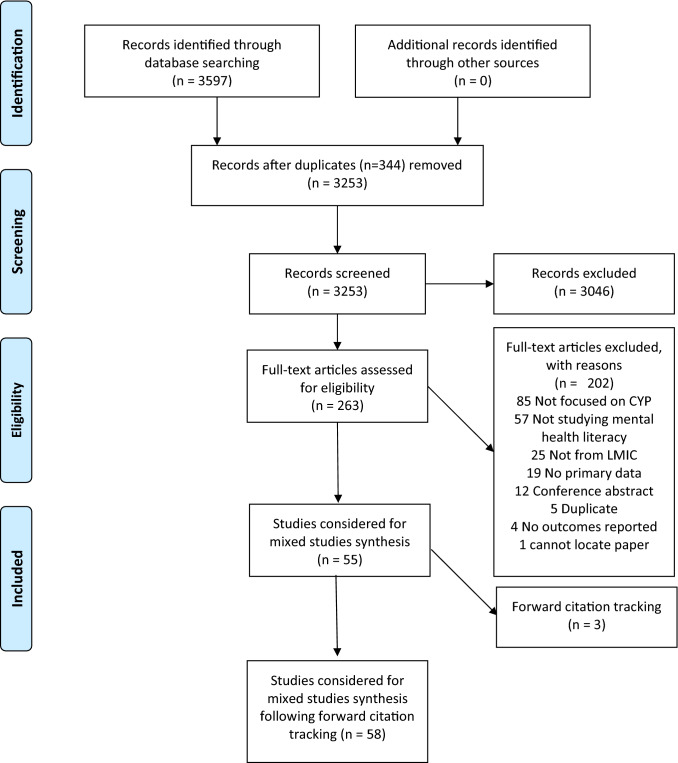
PRISMA flow diagram depicting flow of information screened and reviewed

A PRISMA study flow diagram in Fig. 2 describes how papers were selected for inclusion. 58 papers (41 quantitative, 13 qualitative and 4 mixed methods) representing 52 separate studies were appraised in this review. 36,429 participants with a mean age of 15.3 [10.4–17.4] were included. No study included child populations alone, i.e., up to age 10. With the exception of six papers [31–36], all studies drew their samples from school-going populations.

Studies from upper middle- (*n* = 28) and lower middle-income countries (*n* = 26), as per OECD classification, dominated, with a dearth of published research from the least developed nations (*n* = 2). Across studies, 26 separate LMICs were represented including one study from each Turkey, Iran, Colombia, Sri Lanka, Egypt, Cambodia, Indonesia, Philippines, Papua New Guinea, Uganda, Ghana and Zambia, two studies from each South Africa, Serbia and Pakistan and three from each Vietnam, Jordan (3 same study), Kenya, Malaysia and Brazil. Countries most represented included China (*n* = 6), India (*n* = 4), Nigeria (*n* = 6; 3 same study) and Jamaica (*n* = 5; 3 same study). Four studies conducted internationally compared MHL between high-income and low-income settings [37–40] and one compared MHL between two LMICs [41]. The remainder of included papers were single country studies (Table 1).

**Table 1:** Study characteristics

Reference and country (Author last name, year)	Study design (data collection methods)	Measures of mh literacy /knowledge	Sample (*N*; *n*, % female)	Sample age (mean, [SD, age range])	AIM
Upper Middle-Income Countries					
Abdollahi et al. 2017 *Malaysia*	Survey design—cross-sectional	Attitudes Toward Seeking Professional Psychological Help Scale (ATSPPHS; Fischer and Farina, 1995)	475; 232 (48.8%)	17.29 [3.18, 15–21]	To examine attitudes towards seeking professional psychological help and explore relationships with personality attributes
Ibrahim, Amit et al. 2019 *Malaysia*	Survey design- cross-sectional	Mental Help Seeking Attitude Scale (MHSAS; Hammer et al. 2005) Depression Literacy Scale (Griffiths et al. 2005) Self-stigma of Seeking Help Scale (SSOSH; Vogel et al. 2006) General Help Seeking Questionnaire (GHSQ; Wilson et al. 2005) Beliefs Toward Mental Illness (BMI; Hirai and Clum, 2000)	202; 137 (67.8%)	17.03 [3.36, 13–25]	To examine the factors associated with mental help-seeking attitude among students from low-income households and residing in low-income settings. Differences in beliefs toward mental illness, stigma and help-seeking attitudes among university and secondary school students were investigated
Ibrahim, Mohd Safien et al. 2020 *Malaysia*	Quasi-experimental design	Mental Help Seeking Attitude Scale (MHSAS; Hammer et al. 2005) Depression Literacy Scale (Griffiths et al. 2005) Self-stigma of Seeking Help Scale (SSOSH; Vogel et al. 2006) General Help Seeking Questionnaire (GHSQ; Wilson et al. 2005) Beliefs Toward Mental Illness (BMI; Hirai and Clum, 2000)	101; 61 (60%)	14.61 [1.39, 13–17]	The study aimed to demonstrate the efficacy of the Malaysian Depression Literacy Program immediately after program delivery and at 3-month follow-up among adolescents from low-income populations demonstrating elevated depression levels
Suttharangsee et al. 1997 *Thailand*	Qualitative—ethnonursing	N/R	23; 13 (56%)	17 [N/R, N/R] ^‡‡^	To assess views about what constitutes mental health and beliefs about factors for achieving and maintaining positive mental wellbeing
Chan and Petrus Ng 2000 *China (comparison with Hong Kong data)*	Survey design	Opinion About Mental Illness In Chinese Community (OMICC; Cohen & Struening, 1963) – adapted for this study	China 789; 477 (60.4%) Hong Kong 2,223, 1196 (53.8%)	16.82 [1.17, 12–18] Guangzhou 15.94 [1.20, 12–18] Hong Kong	To explore attitudes and beliefs about mental illness
Chen et al. 2014 *China*	Survey design	Self-Stigma of Help Seeking Scale (SSOHSS; Vogel et al., 2006), Perceived Devaluation-Discrimination Scale (PDDS; Link, 1987), Attitudes Toward Seeking Professional Psychological Help Scale (ATSPPHS; Fischer and Farina, 1995), Willingness To Psychological Help-Seeking Scale for Middle School Students (WTPHSS; Xu, 2008)	251; 146 (58%)	14.22 [1.46, 11–17]	To explore willingness to seek professional help for psychological problems, association with number of mental health problems and assess whether self-stigma and public stigma influences this relationship
Tan et al. 2017 *China*	Survey design—three-phase stratified cluster design	Suicide Attitudes Inventory (Xiao et al. 1999) – based on the Suicide Attitude Questionnaire (Domino, 1982) and validated for Chinese populations. (Domino et al. 1982)	6568; 3168 (48%)	13.94 [1.83, 9–18]	To evaluate attitudes towards suicide and suicidal ideation and explore the relationship with mental health status
Teo, Shi et al. 2020 *China*	Survey design – cross-sectional	Questionnaire measuring perceptions of internet for help-seeking- origin, development or psychometric properties not reported	1216; 614 (50.5%)	14.21 [1.28, 11–17]	To predict intention to use cyber-counselling among Chinese adolescents using an extended theory of planned behaviour model
Yu, Lou et al. 2019 *China*	Multi-method qualitative study including photovoice, community mapping and focus group discussions	N/R	90; 44 (48.8%)	17.4 [1.3; 15–19]	To understand the factors that facilitate and hinder disadvantaged adolescents from obtaining the health information and services they need to secure good health
Yamaguchi et al. 2014 *China (comparison with Japan and South Korea)*	Survey design – cross-sectional with between group comparison	Selected items from the UK Pinfold Questionnaire (Pinfold et al., 2003)	1011; 489 (48.4%)	N/R [N/R, 13–14]	To examine factors that influence stigmatising beliefs about mental health problems
Eskin 1999 *Turkey (comparison with Sweden)*	Survey design	Questionnaire developed specifically for the study – no information about development or adaptation given	146; 71 (48.6%) Turkey 108; 49 (45.4%) Sweden	16.1 [0.8, 14–18] Turkey 17.2 [0.9, 16–20] Sweden	To explore attitudes and beliefs about prognosis, treatment and perceptions of mental illness and suicide
Yilmaz-Gozu et al. 2013 *Turkey*	Survey design – cross-sectional	Attitude Toward Seeking Professional Help Scale (ATSPHS; Ozbay, Yazıcı, Palancı and Koc, 1999)	342; 198 (58%)	N/R [N/R, N/R]	To explore help-seeking attitudes including recognition of the need for help, stigma tolerance, interpersonal openness, confidence in mental health professionals and psychological distress
Essau, Olaya et al. 2013 *Iran*	Survey design	Mental Health Literacy for Depression questionnaire (Jorm et al., 1997)—original questionnaire was adapted and translated from English to Farsi	1984; 1006 (50.7%)	14.49 [1.7, 12–17]	To investigate recognition of depression, beliefs about causation and treatments and views about self-help for preventing depression in relations to socio-demographic characteristics and exposure to depression
Aggarwal, Berk et al. 2016 *South Africa*	Survey design – pre-intervention	Semi-structured questionnaire – development or psychometric properties not reported	1999: 950 (47.5%)	15.78 [1.78, 13–22]	To explore knowledge and recognition of depression symptoms and preferences for seeking support
Shilubane et al. 2014 South Africa	Qualitative	N/R	56; 30 (53.6%)	N/R [N/R, 13–19]	To assess attitudes towards suicide, perceived risk factors and signs of potential suicide risk, awareness of available mental health care and beliefs about preventing suicide
Morais et al. 2012 *Brazil* *In Portugeuese*	Survey design	Access to Mental Health Care to Children – (AMHC; Kappler et al., 2004) – adapted for this study	1,168; 619 (53%)	15.80 [1.68, 10–21]	To explore the concepts of mental health and welling and understand self-help strategies to improve wellbeing
Fukuda et al. 2016 *Brazil*	Survey design – convenience sampling among three different schools, descriptive and correlational	Access to Mental Health Care to Children – (AMHC; Kappler et al., 2004) – adapted and validated by Aquina-Morais et al., 2014)	1030; 540 (52.4%)	15.3 [1.8, 8–21]	To explore attitudes towards professional psychological help, barriers to help-seeking comparing clinical and non-clinical samples
Jenkins, Sanchez et al. 2019 *Mexico* *In Spanish*	Mixed methods comprising quantitative (socio-demographic questionnaire and standardised symptom scales) and qualitative (in-depth ethnographic interviews, observation) components	N/R	35; 20 (57.1%)	15.9 [0.7, 15–17]	To generate an ethnographically informed understanding of contexts and processes that shape the emotional wellbeing and mental health of adolescents
Gonzalez-Fuentez et al. 2016 *Mexico* *In Spanish*	Mixed methods comprising quantitative survey derived from qualitative analysis	Scale development of psychological well-being	1635; 856 (52.35)	N/R [N/R, 14–20]	To qualitatively evaluate the meaning of psychological wellbeing for adolescents and design and validate a scale to measure this construct
Paula et al. 2009 *Brazil*	Qualitative—focus groups and key informant interviews with families and service providers	N/R	46; 28 (60.1%)	13.7 [N/R, 11–16]	To explore views about the causes of emotional and behavioural problems and experiences of seeking care
Gomez-Respetro et al. 2021 Colombia	Survey design- nationally representative sample from household survey	Single item: self-report inquiring whether an adolescent had been told they had a mental health problem by a professional	1754; N/R	N/R [N/R, 7–18]	The aim was to determine potential factors associated with whether mental disorders and problems are recognized in the Colombian population, specifically adolescents
Jackson 2007^§¶^ *Jamaica*	Survey design	Opinions about Mental Illness, (OMI; Stuening & Cohen, 1963), Attitudes Toward Seeking Professional Psychological Help Scale, (ATSPPHS; Fischer & Turner, 1970 “Where Do You Go To For Help” Questionnaire –scale development by the author for this study, no psychometric properties reported	339; 193 (57%)	17.18 [0.76, 15–19]	To explore attitudes towards seeking professional psychological help, beliefs about mental illness and help-seeking preferences and investigate in relation to symptoms of mental illness
Maloney, Abel et al. 2020 *Jamaica*	Survey design – cross-sectional	Attitudes Toward Seeking Professional Psychological Help (ATSPPHS; Fischer & Turner, 1970) Survey adapted from Ben-Zeev et al., 2017 – no psychometric properties reported	56; 32 (57%)	N/R [N/R, 10–19]	To conduct a feasibility study to determine the viability of deploying digital mental health resources in Jamaica to adolescent populations, with a particular focus on identifying variations in infrastructure and preferences between rural and urban populations
Williams 2012^§^ *Jamaica*	Survey design – cross-sectional	“Where Do You Go To For Help” Questionnaire –scale development by the author for this study, no psychometrics reported	339; 193 (57%)	17.18 [0.76, 15–19]	To explore preferences for professional help-seeking and beliefs about treatment usefulness for different mental health problems
Williams 2014^§^ *Jamaica*	Survey design – cross-sectional	Attitudes Toward Seeking Professional Psychological Help (ATSPPHS; Fischer & Turner, 1970) modified by Atkinson and Gim, 1989; Opinions About Mental Illness Scale (OMI; Cohen & Struening, 1962)	339; 193 (57%)	17.18 [0.76, 15–19]	To examine the contribution of beliefs about aetiology, beliefs about mental illness and causation and socio-demographic factors to attitudes towards psychological help-seeking
Williams 2013^§^ *Jamaica*	Survey design – cross-sectional	Attitudes Toward Seeking Professional Psychological Help (ATSPPHS; Fischer & Turner, 1970)	339; 193 (57%) Jamaican 81; 12 (15%) African American	17.18 [0.76, 15–19] Jamaican 15.98 [1.13, 14–18] African American	To evaluate attitudes towards psychological help-seeking and draw comparison with a high-income country
Cankovic et al. 2013 *Serbia* *In Serbian*	Survey design	Suicide Opinion Questionnaire (Domino, 1996)	254; N/R (N/R)	N/R [N/R, 13–19]	To explore attitudes towards suicide
Pejovic-Milovancevic et al. 2009 *Serbia*	Pre and post-test design	Opinion about Mental Illness (OMI; Stuening & Cohen, 1963)	63; N/R (N/R)	N/R [N/R, 15]	To evaluate awareness of mental health-related issues and assess stigmatising behaviours prior to receiving mental health awareness sessions
Lower Middle-Income Countries					
Attygalle et al. 2017 *Sri Lanka*	Survey design—descriptive cross-sectional	Australian National Survey on Mental Health Literacy (Reavley & Jorm, 2011) – modelled on this questionnaire	1002; 421 (42%)	14.00 [0.94, 13–16]	To explore recognition of mental health problems, attitudes towards seeking professional help and views about potential treatment outcomes
Nastasi and Borja 2015^#^ Chapter 6 Adelson et al *India*	Qualitative—focus groups and ecomap activities	N/R	37; 37 (100%)	N/R, [N/R, 12–20]	To explore stressors and protective factors for psychological wellbeing
Sharma et al. 2017 *India*	Survey design—cross-sectional	Australian National Survey on Mental Health Literacy (Reavley & Jorm, 2011) – adapted and modified for Indian context	354; 168 (47.5%)	N/R [N/R, 13–17]	To evaluate depression recognition, help-seeking intentions, and beliefs about interventions, causes, risk factors, outcomes, and stigmatizing attitudes
Parikh, Michelson et al. 2019 *India*	Multi-method qualitative (stakeholder interviews and focus group discussions)	N/R	191; 112 (58.7%)	N/R [N/R, 11–17]	To elicit the views of diverse stakeholders including adolescents in two urban settings in India about their priorities and preferences for school-based mental health services
Shadowen et al. 2019 *India*	Mixed methods – quasi-experimental design with qualitative inquiry	N/R	15; N/R (N/R)	N/R; [N/R, 12–14]	To measure the impact of an after-school resilience-building program for a group of marginalized Indian schoolchildren in rural farming villages of Tamil Nadu, India
Afifi 2004 *Egypt*	Survey design – multistage stratified random sampling	Attitude Towards Suicide Scale (ATSS; Eskin, 2004)	1621; 801 (49.4%)	15.77 [1.36, 14–19]	To evaluate attitudes towards suicide and the relationship between ideation and attempts
Nguyen et al. 2013 *Vietnam*	Multi-method qualitative (stakeholder interviews, key informant interviews, focus groups)	N/R	138; 83 (60%)	N/R [N/R, 15–18]	To explore perceptions of mental health and views about what are the risks for mental health problems alongside identifying stakeholder strategies to improve mental health
Nguyen, Dang et al. 2020 *Vietnam and Cambodia (Cambodia – least developed country class)*	Experimental evaluation (pre-post randomised design) in Vietnam (study 1) and preliminary efficacy study in Cambodia (study 2)	Mental Health Knowledge and Attitude Test (Kutcher & Wei, 2017)	Study 1: 2539; 1320 (52%) Study 2: 275; 171 (62%)	N/R [N/R, N/R] Study 1: 15 (median) Study 2: 16 (median)	To evaluate the efficacy of an evidence-based MHL program in Vietnam adapting an existing program and assess portability in a pilot efficacy study in neighbouring Cambodia
Thai, Vu et al. 2020 *Vietnam*	Survey design- cross-sectional cluster sampling	Mental Health Literacy Scale (MHLS; O’Connor & Casey, 2015) General Help-Seeking Questionnaire (GHSQ; Wilson et al. 2005)	1075; (56.2%)	N/R [N/R, N/R]	To evaluate the level of mental health literacy and help-seeking preferences in high school students in Ho Chi Minh City, Vietnam
Willenberg, Wulan et al. 2020 *Indonesia*	Qualitative – focus group discussions	N/R	86; 41 (47.7%)	17 [N/R; 16–18] ^††^	To understand conceptualisations and perceived determinants of mental health from the perspective of Indonesian adolescents
Estrada, Nonaka et al. 2019 *Phillipines*	Mixed methods comprising quantitative (cross-sectional survey) and qualitative (in-depth interviews) components	N/R	183; 58 (33.9%) Study 1: 171, Study 2: 12	N/R [N/R, N/R]	To describe the prevalence of suicidal ideation and behaviours, attitudes towards suicide among adolescent learners in alternative education. Additionally, relationships between suicidal ideation, behaviours, participant characteristics, attitudes and alternative learning environment were evaluated
Dardas, 2018^†¶^ *Jordan*	Survey design—nationally representative, school-based sample	Depression Etiological Beliefs Scale (Samouilhan & Seabi, 2010; Wadian, 2013), Depression treatment seeking scale (Barney, Griffiths, Jorm, & Christensen, 2006) Depression Stigma Scale (Griffiths et al., 2008)	1389; 820 (59%)	N/R [N/R, 12–17] ^§§^ 168 (7.0%) 12 259 (11%) 13 357 (15%) 14 396 (17%) 15 839 (36%) 16 312 (14%) 17	To explore beliefs about depression causation, stigmatising beliefs and the likelihood of seeking help in relation to depression symptoms
Dardas et al. 2018^†^ *Jordan*	Survey design – pilot study to assess feasibility of obtaining nationally representative sample	N/R	88; 35 (40%)	16 [0.5, 15–17	To examine the methodology for research examining depression stigma and attitudes towards professional help-seeking in relation to depression severity
Dardas, Shoqirat et al. 2019 *Jordan*	Qualitative – focus group discussions	N/R	92; 56 (61%)	15 [N/R, 14–17}	To capture adolescents’ experiences of depression, identify perceived contributing factors and assess attitudes towards depression interventions
Rahman, Mubbashar et al. 1998 *Pakistan*	Quasi-experimental design – control group with no randomisation to a school mental health programme	Measure designed for this study – no development or validation reported	100; 50 (50%) intervention, 50 (50%) control	N/R [N/R, 12–16]	To evaluate awareness of mental health-related problems prior to receiving mental health awareness programme
Khalil et al. 2020 *Pakistan*	Survey design-cross-sectional	Paediatric Self-Stigmatization Scale (PaedS) Kaushik, A., Papachristou, E., Dima, D., Fewings, S., Kostaki, E., Ploubidis, G.B., Kyriakopoulos, M., 2017)	110; 55 (50%)	N/R [N/R, 8–17]	To measure the stigma associated with mental illness in children with a variety of psychiatric diagnoses
Callan et al. 1983 *Papua New Guinea (comparison with Australian data)*	Survey design- correlational analysis and content analysis of free text responses	Scale developed for this study from existing measures (Brockman and D'Arcy, 1978; Trute & Loewen, 1978; Dieman et al., 1973; Nunnally, 1961)	Papua New Guinea 133; 57 (42.3%) Australia 144; 51 (35.4%)	17 [N/R, N/R] males 16 [N/R, N/R] females (PNG students only)	To explore attitudes and beliefs about mental illnesses and treatment
Aluh et al. 2018 *Nigeria*	Survey design – descriptive cross-sectional	Friend in Need Questionnaire (Burns and Rapee, 2006)	285; 143 (50.20%)	14 [N/R, 12–18] ^‡‡^	To explore knowledge and recognition of depression and help-seeking behaviours
Bella et al. 2012* *Nigeria*	Survey design—qualitative thematic analysis of free text responses	N/R	164; 85 (52%)	14.8 [1.8, 10–18]	To explore views about mental illness, causation, manifestations and treatment
Bella-Awusah et al. 2014 *Nigeria*	Quasi-experimental design -two group pre-test–post-test control group design without randomisation	World Psychiatric Association Anti-stigma Schools Project/UK Pinfold Questionnaire (Pinfold et al. 2003) – adapted for this study	154; approximately 50% were female in intervention (51.9%) and control (50.7%)	15.3 [1.6, 10–18] intervention 14.3 [2.0, 10–18] control	To assess the impact of a school-based mental health awareness programme in mental health literacy and stigmatising beliefs
Dogra et al. 2012* *Nigeria*	Survey design – cross-sectional	World Psychiatric Association Anti-stigma Schools Project in Canada (World Psychiatric Association, 2000) – adapted to Nigerian context	164; 85 (52.1%)	14.8 [1.8, 10–18]	To explore knowledge and attitudes towards mental health and illness including stigmatising beliefs
Oduguwa et al. 2017 *Nigeria*	Quantitative—quasi-experimental with an intervention and control group	UK Pinfold Questionnaire (Pinfold, 2003) –adapted, translated and validated for use in Nigeria	205; 95 (47%)	14.91 [1.3, 10–17]	To explore attitudes, knowledge and stigmatising beliefs about mental illness prior to receiving mental health awareness training
Ola et al. 2015 *Nigeria*	Survey design – participants were children whose parents were psychiatric inpatients in the mental health unit of the Lagos State University Teaching Hospital	Selected items from previous studies (Adewuya and Makanjuola, 2008; Ani et al., 2011) – no psychometric properties reported	67; 43 (64%)	13.34 [2.87, 7–18]	To explore beliefs about mental illness and perceived risks for mental distress in children whose parents have mental illness
Ronzoni et al. 2010* *Nigeria*	Cross-sectional survey with qualitative analysis of free text responses	World Psychiatric Association’s anti-stigma schools project in Canada (World Psychiatric Association, 2000)/UK Pinfold Questionnaire (Pinfold et al., 2003)	164; 81 (49.4%)	N/R [N/R, 12–18] ^§§^ 24 (14.7%) 10–12 51(31.1%) 13–14 52 (31.2%) 15–16 36 (22%) 17–18	To explore views about mental illness, mental health problems, stigmatising beliefs and beliefs about causes
Ndetei, Mutiso et al. 2016 *Kenya*	Survey – random school clusters selected from 2 districts in Kenya and 23 schools from each cluster selected using simple random sampling, students in classes in selected clusters participated	Self-Stigma of Mental Illness Scale (SSMIS; Corrigan et al., 2006) – stereotype agreement subscale used, translated into local languages	4585; 2290 (49.9%)	10.4 [2.5, 5–21]	To investigate stigmatizing beliefs about mental illness and examine the relationship with socio-demographic factors
Secor-Turner, Randall et al. 2016 *Kenya*	Qualitative	N/R	64; 32 (50%)	16.2 [N/R, 12–26]	To evaluate perceived barriers and facilitators of health in a cultural context
Tamburrino et al.2020 *Kenya*	Qualitative interviews	N/R	7; N/R	N/R [N/R, 14–17]	To explore how youth stakeholders conceptualize mental illness, contributing factors and required supports for disadvantaged young people in Kenya
Glozah 2015 *Ghana*	Qualitative study using semi-structured interview	N/R	11; 6 (54.5%)	16.86 [N/R, N/R]	To explore perspectives of interpersonal support for personal wellbeing
Least Developed Countries					
Nalukenge, Martin et al. 2018 *Uganda*	Qualitative interviews	N/R	19; 11 (58%)	14 [N/R, 12–17] ^††^	To explore perspectives and beliefs about mental illness causation among children with positive HIV status
Dhadphale 1979 *Zambia*	Survey design	N/R	69; 69 (100%)	N/R [N/R, 14–19]	To explore perspectives of spirit possession, recognition of treatment need and help-seeking

*Using the same data

^†^Using the same data

^§^Using the same data

^¶^Dissertation

^#^Book/monograph

**N/R = not reported

^††^Median and/or IQR reported

^‡‡^Mode reported

^§§^Proportions reported in age bands

### Quality appraisal

The quality of studies ranged from 0 (no criteria met) to 100% (all criteria met) on the MMAT. The majority of qualitative studies achieved scores of 100% while fewer studies utilising other designs achieved high scores on quality. Overall, randomised trials and quasi-experimental designs were of poorer quality. Studies employing descriptive quantitative designs were predominantly moderate quality. Variation in quality was evident; risk of selection and measurement bias were identified in many studies, specifically in use of instruments not validated for target populations. Quality appraisals are detailed in Table 2.

**Table 2:** Quality Appraisal

	Screen		Qualitative					Quality Score
	1	2	Appropriate methods	Adequate data collection	Findings derived from data	Interpretation substantiated	Methodological coherence	%, Score
Suttharangsee et al. 1997	✓	✓	✓	✓	✓	✓	✓	100 Strong
Nalukenge et al. 2018	✓	✓	✓	✓	✕	✕	✕	40 Weak
Adelson et al. 2015	✓	✓	✓	✓	✓	✓	✕	80 Strong
Paula et al. 2009	✓	✓	✓	✓	✓	✓	✓	100 Strong
Glozah 2015	✓	✓	✓	✓	✓	✓	✓	100 Strong
Ronzoni et al. 2010	✓	✓	✓	✓	✓	✓	✓	100 Strong
Shilubane et al. 2014	✓	✓	✓	✓	✓	✓	✓	100 Strong
Secor-Turner et al. 2016	✓	✓	✓	✓	✓	✓	✓	100 Strong
Nguyen et al. 2013	✓	✓	✓	✓	✓	✓	✓	100 Strong
Tamburrino et al. 2020	✓	✓	✓	✕	✓	✓	✓	80 Strong
Parikh et al. 2019	✓	✓	✓	✓	✓	✓	✓	100 Strong
Yu et al. 2019	✓	✓	✓	✓	✓	✓	✕	80 Strong
Dardas et al. 2019	✓	✓	✓	✓	✓	✓	✓	100 Strong
Willenberg et al. 2019	✓	✓	✓	✓	✓	✓	✓	100 Strong

	Screen		Quantitative randomized controlled trials					
	1	2	Appropriate randomisation	Comparable baseline groups	Complete outcome data	Blind outcome assessors	Participant intervention adherence	
Rahman et al. 1998	✓	✓	✕	✓	✓	✕	✕	40 Weak
Nguyen et al. 2020	✓	✓	✕	✓	✓	✕	✓	60 Mod-Weak

	Screen		Quantitative non-randomized trials					
	1	2	Representativeness	Appropriate measures	Complete outcome data	Confounders considered	Intervention administration	
Pejovic-Milovancevic et al. 2009	✕	✕	✕	✕	✓	✕	✕	20 Weak
Callan et al. 1983	✓	✓	✕	✕	✕	✕	✓	20 Weak
Oduguwa et al. 2017	✓	✓	✕	✓	✕	✓	✓	60 Mod-weak
Yamaguchi et al. 2014	✓	✓	✕	✕	✕	✕	✓	20 Weak
Bella-Awusah et al. 2014	✓	✓	✓	✕	✓	✓	✓	80 Strong
Ibrahim et al. 2020	✓	✓	✕	✓	✓	✕	✕	40 Weak

	Screen		Quantitative descriptive					
	1	2	Sampling Strategy	Representativeness	Appropriate measures	Risk of non-response bias	Appropriate statistical analysis	
Aggarwal et al. 2016	✓	✓	✕	✕	✕	✕	✕	0 Weak
Cankovic et al. 2013	✓	✓	✕	✕	✕	✕	✓	20 Weak
Dhadphale 1979	✓	✕	✕	✕	✕	✕	✕	0 Weak
Tan et al. 2017	✓	✓	✓	✓	✓	✓	✓	100 Strong
Ola et al. 2015	✓	✓	✓	✕	✓	✕	✓	60 Mod-weak
Ndetei et al. 2016	✓	✓	✓	✕	✓	✕	✓	60 Mod-weak
Fukuda et al. 2016	✕	✕	✓	✓	✓	✕	✓	80 Strong
Eskin 1999	✕	✕	✓	✕	✓	✓	✓	80 Strong
Jackson 2007	✓	✓	✓	✕	✓	✕	✓	60 Mod-weak
Dogra et al. 2012	✓	✓	✓	✕	✓	✕	✓	60 Mod-weak
Dardas et al. 2018	✓	✓	✓	✓	✓	✓	✓	100 Strong
Dardas 2018	✓	✓	✓	✓	✓	✓	✓	100 Strong
Chen et al. 2014	✓	✓	✓	✓	✓	✕	✓	80 Strong
Chan & Petrus Ng 2000	✓	✓	✓	✓	✕	✕	✓	60 Mod-weak
Bella et al. 2012	✓	✓	✓	✓	✕	✕	✓	60 Mod-weak
Afifi 2004	✓	✓	✓	✓	✓	✕	✓	80 Strong
Attygalle et al. 2017	✓	✓	✓	✓	✓	✕	✓	80 Strong
Abdollahi et al. 2017	✓	✓	✓	✕	✓	✕	✓	60 Mod-weak
Yilmaz-Gozu 2013	✓	✓	✓	✕	✓	✕	✓	60 Mod-weak
Williams 2014	✓	✓	✓	✕	✓	✕	✓	60 Mod-weak
Williams 2012	✓	✓	✓	✕	✕	✕	✕	20 Weak
Morais et al. 2012	✓	✓	✓	✓	✓	✓	✓	100 Strong
Sharma et al. 2017	✓	✓	✓	✕	✕	✓	✕	40 Weak
Essau et al. 2013	✓	✓	✓	✓	✓	✓	✓	100 Strong
Aluh et al. 2018	✓	✓	✕	✓	✓	✓	✓	80 Strong
Williams 2013	✓	✓	✓	✕	✓	✕	✕	40 Weak
Gomez-Restrepo et al. 2021	✓	✓	✓	✓	✕	✕	✓	60 Mod-weak
Khalil et al. 2019	✓	✕	✓	✓	✓	✕	✕	60 Mod-weak
Thai et al. 2020	✓	✓	✓	✕	✓	✓	✓	80 Strong
Maloney et al. 2020	✓	✓	✕	✕	✓	✕	✓	40 Weak
Ibrahim et al. 2019	✓	✓	✕	✕	✓	✕	✓	40 Weak

	Screen		Mixed methods					
	1	2	Appropriate rationale	Study components integration	Adequate output interpretation	Divergences addressed	Quality criteria adherence	
Gonzalez-Fuentes et al. 2016	✓	✓	✓	✓	✓	✕	✓	80 Strong
Jenkins et al. 2019	✓	✓	✕	✕	✕	✕	✕	0 Weak
Estrada et al. 2019	✓	✓	✓	✓	✓	✕	✓	80 Strong
Shadowen 2019	✓	✓	✕	✓	✕	✕	✕	20 Weak

2 quality score: Weak ≤ 50%, Moderate-weak 51–65%, Moderate-strong 66–79%, Strong > 80%

### MHL components

#### Ability to recognise mental illnesses

Evidence of CYP ability to recognize mental illnesses in others was primarily derived from surveying convenience samples of school-going CYP and responses were varied [41–51]. Less than half of populations examined responded correctly to questions about common signs and symptoms, risk factors and aetiology, effectiveness of pharmacological and non-pharmacological treatments and the prognosis of mental illnesses including outcomes. Proportions of adolescent samples (range *n* = 354–1999) accurately endorsing indicative features of mental illnesses ranged from 1.5 to 49.9% [43–45]. Mean scores of depression literacy in separate samples assessed at different times but drawn from similar populations in Malaysia (range *n* = 101–202) varied from 5.01 to 12.67 on a 21 point scale [48, 49]. Vietnamese adolescents (*n* = 1075) had mean scores on general mental health literacy ranging from 2.49 to 2.67 (5 point subscales) on assessments of recognition, knowledge of risk factors and self-treatment [51].

Vignette methodologies used in large populations (range *n* = 285–1999) found that 83.6% of secondary school students in Sri Lanka accurately detected depression compared to 82.2% in India, 50% in Iran and 4.8% in Nigeria [42, 43, 45, 46]. Just one study evaluated CYP knowledge of other diagnoses (psychosis and social phobia) and demonstrated identification accuracy of 68.7% and 62.1% respectively [45]. Young people in included studies who displayed symptoms of mental illness, most commonly depression, but were undiagnosed and untreated, demonstrated limited ability to recognize their own mental illness [52–54]. However, evidence was obtained from moderately weak methods employed in quantitative designs. Between 8.3 and 50% of those with clinically significant symptoms in nationally representative cohorts (range 1754–2349) recognized having a problem that would warrant treatment [52, 53].

Eliciting words or phrases attributed to someone with mental illness, with the purpose of evaluating knowledge and by default, misconceptions about mental illness, was used in a minority of qualitative and quantitative studies. These studies highlighted CYP beliefs about the manifestation of mental illness in the form of visual or behavioural indicators which could be observed externally [40, 55, 56]. Five studies included evidenced that young people (range *n* = 19–1168) also attributed a range of typical symptoms to mental illnesses [33, 57–60]. Symptoms included sad mood, anxiety, apathy, social withdrawal, insomnia, decreased ability to think and lack of appetite. Despite this, many used derogatory terms to describe mental illnesses and held several stigmatizing views [39–41, 55]. Several of these textual descriptive analyses of the content of stigmatizing terms were derived from lower quality studies; however, there was further stronger evidence of pervasive stigmatised views (see section below).

Robust quantitative data from samples optimized for representativeness and measuring the relationship between age and MHL, evidenced that older participants and those with more education years tended towards better recognition than younger participants [46, 48, 53]. Females also tended towards improved recognition, though few studies examined sex differences [42, 46]. Social factors were also significant and higher socio-economic status and better recognition were linked in two studies [45, 53]. Similarly, geographic region may be important. Comparisons between countries regarding knowledge and treatments found no differences in literacy levels between high and lower income settings [37, 39, 57]. However, preliminary quantitative and qualitative data from exploratory analyses indicate within country differences may exist such that familiarity with psychiatric terminology and recognition of mental illnesses, is better in urban settings than rural [47, 56]. The quality of included studies was uneven, stronger evidence supported assertions that disorder recognition varied between settings and was mainly limited to depression. Weaker evidence was found that recognition and knowledge varied temporally, no studies assessed recognition longitudinally.

#### Knowledge of causes or risk factors for mental health problems

Quantitative, survey data across Vietnam, Jordan, Iran, India, Papua New Guinea, Nigeria and China illustrate specific misconceptions about the origins of mental illnesses alongside beliefs that mental illnesses are biopsychosocial in nature [38, 39, 44, 46, 47, 61]. Evidence indicates beliefs that mental illnesses are caused by weaknesses of character among 25–50% of convenience and representative samples [44, 59, 61]. Immoral behaviour was prominent among adolescent beliefs though perceptions of what constitutes immorality are, to an extent, socially and culturally determined [47]. Specific misdeeds varied between studies, e.g. familial jealousy, envy, adultery, theft, polygamy and interpersonal conflict were viewed as immoral in separate studies [47, 54, 59].

Religious, spiritual and supernatural explanations for mental illnesses were also pervasive within included studies. The majority of research examined the proportions of accurate causal attributions among possible answers in adolescent populations obtained using both purposive or probability sampling. Evidence was moderately strong that punishment for sin or test from God was considered causative, reported in between 10 and 35% of cross-sectional analyses [34, 44, 46, 54, 61]. There was also a perception that mental illness could be caused by a lack of faith or religion in qualitative analyses [54, 56]. Supernatural explanations for illnesses were endorsed by between 38 and 64% of adolescents in included studies [47, 50, 61]. Witchcraft, wizardry, evil spirits and sorcery were prominent interpretations [34, 39, 46, 50, 62] though the quality of included studies was mixed and older analyses may not represent present-day causal beliefs.

Confusion between physical and mental illnesses was highlighted in one study, particularly among younger participants [63], again evidence was not current and considered weak. However, there were repeated findings from quantitative designs that attributing mental illness to physical aetiology was prominent [33, 34, 46, 47, 55, 59, 63] and generally, evidence strongly supported varied physical explanations. Responses obtained to open questions in cross-sectional surveys about what causes mental illness or what it could reveal showed adolescents believed that fever, hypertension, malnutrition, head trauma, and stomach aches and pains, cerebral palsy, epilepsy and HIV variously contributed to mental illnesses [33, 46, 47, 55, 59]. Two studies provided evidence that adolescents believed mental illnesses were contagious [34, 63].

Normalising beliefs and evidence of wider illness conceptualisations were prominent in some CYP cohorts. Substantial proportions (between 60 and 72%) of large, diverse, adolescent cohorts in China, Iran, and India (range *n* = 354–1954) believe social factors and stressful life events are causative [38, 44, 46]. While a range of familial and societal stressors including family illness, abuse, trauma, bereavement, discrimination and poverty were identified, a strong theme emerged in both qualitative and quantitative data that upbringing and poor parenting are considered to cause mental illnesses [33, 38, 46, 61, 64]. Qualitative inquiry expounded this theme illuminating perceived causal factors including protective and autocratic parenting, lack of family communication and support, family history of mental illness, parental conflicts and poor family socio-economic situation [54], parental pressure to perform in academia [56] and lack of warmth and intimacy in familial relationships [65]. Beliefs that mental illnesses are biological or genetic in nature were identifiable in three quantitative studies [44, 46, 61]. These causes were reported by proportionately less CYP compared to other factors attributed to the onset of mental illness (10–33%).

#### Knowledge and beliefs about self-help interventions

Evidence of self-help strategies to enhance wellbeing [32, 56, 65–67] and manage mental health problems [68–70] is derived mainly from qualitative studies investigating CYP perspectives. Proactive and protective strategies identified from these studies included cognitive processing of stressful events [58], positive thinking [32] having good manners and behaving well [66], and community participation [56]. Stress avoidance was recognised as an important strategy as well as focusing on behaviours that enhance self-esteem and empowerment [67] and obtaining practical advice from others [68]. Cross-sectional analyses of quantitative data described self-help strategies CYP believed helpful for preventing depression [44, 46]. Beneficial practices endorsed by more than half of samples include meditation, relaxation training, using self-help books, increased physical activity, avoiding illicit and mood-altering substances, and taking vitamins. Interestingly, among both samples taking vitamins was considered more beneficial than taking antidepressants (54% vs 27–30%) [44, 46].

Exploratory qualitative analyses among Mexican and Brazilian CYP found a preference for dealing with problems alone, for example, seeking solace in their own rooms was considered important for processing difficult emotions like sadness, anger and stress [32, 65, 69]. Where CYP did express a preference in eliciting help from others, important sources generated from qualitative analyses included parents [32, 58, 66, 71] mothers in particular [32, 66], family members [32, 42, 61, 71, 72], peers [32, 58, 66, 71], siblings [43, 58, 71] teachers [58], church leaders [70], and neighbours/community members [32]. Two of these studies were appraised as weaker evidentiary sources [65, 71].

Members of immediate social networks were often the preferred option for advice and counsel about personal concerns from both quantitative and qualitative analyses [51, 54, 56, 71]. CYP in included studies appeared to purposely select certain social ties in response to specific problems [58, 73]. Selection also differed between genders; males were more likely to seek support from teachers, family members and friends compared to females [46]. Evidence is mixed regarding whether males or females are more likely to access school counsellors but counsellor gender may be an important factor in choosing whether to seek help for specific problems [73]. Crucially, as relationships with parents were also sometimes viewed as a contributing factor to stress in addition to being important sources of support, qualitative analyses showed that CYP with difficult relationships with parents were unlikely to view, and seek out, parents as a source of support [54] while qualitative evidence from 90 participants indicates support from both teachers and parents is prioritised by younger adolescents [74].

#### Knowledge and beliefs about professional help available

Between 40 and 75% of survey, cohorts believed mental illnesses were treatable by health professionals [34, 45, 50–52, 61, 63, 71, 72, 75] though individual studies varied in quality. Studying preferences more in depth found that formal help was considered comparatively more beneficial than none at all. However, CYP more often stated a preference to seek help from family, friends and traditional healers over mental health professionals [71, 75] though evidence was generated from two exploratory feasibility studies which were among the few that ranked the importance of different sources of professional help. In some studies, formal health services were considered more useful for physical health conditions such as diabetes compared to mental health problems, such as psychosis, depression and social phobia [42, 45, 46]. Among a sample of over 1000 Brazilian CYP those currently receiving or previously in receipt of psychological treatment had greater confidence in its usefulness [76] but overall, misgivings about the effectiveness of professional help were prominent.

Data regarding the perceived usefulness of health professionals and services were primarily derived from cross-sectional analyses using existing help-seeking measures. Health professionals were in the minority of identified sources of help but those that appeared salient included psychologists [42, 44, 46, 52], psychiatrists [42, 52, 61, 62, 75], GP [61, 62, 75] and school counsellors [42, 44, 56, 61, 75]. Less frequently, cardiologists/neurologists [69] and social workers [43] were considered key contacts. Limited understanding of the types of help available and the types of problems that might be treated by professionals emerged. CYP narrowly viewed help as individual counselling or psychotherapy [54, 68] without considering other professionals, types of therapy or formats for delivery. Where CYP were informed about the purpose of professional help, issues with infrastructure and provision were considered prominent barriers, i.e. lack of appropriate services or adequately trained professionals, long waiting times for treatment, limited availability of and resources within health services and health professionals not being interested in mental health problems [61, 69].

CYP instead highlighted the value attributed to religious and spiritual leaders and traditional healers in both qualitative and quantitative analyses [39, 50, 52, 54, 61, 62, 69, 75]. This group of paraprofessionals were often considered more helpful than professionals in formal health services [43, 59, 62] though it is important to recognise that evidence varied in quality. Preliminary evidence pointed to gender differences in views about professional helpfulness and there may be observable differences between males and females in help-seeking choices [46], but inquiry in this area is limited.

#### Factors and attitudes that facilitate recognition and help-seeking

Various facets of stigma were investigated, widely demonstrating that stigma levels are significant and shared by large proportions of CYP. Survey and pre-intervention data from separate studies in China, India, Iran, Pakistan, Vietnam and Cambodia found sizeable segments of CYP cohorts view people with mental health problems as dangerous/violent or frightening; proportions ranged from 25 to 80% [38, 40, 41, 44, 46, 51, 63]. Three separate studies recruiting smaller convenience samples for descriptive purposes or pre-intervention baseline analysis (range *n* = 67–205) in separate regions of Nigeria found high rates of similarly stigmatising beliefs [34, 47, 50]. For example, Dogra et al. [47] found 25% of respondents believed schizophrenia a split personality and a further 60% were unsure. People with mental illnesses are considered difficult to talk to [38, 47, 50, 77]; but furthermore, being associated with someone with a mental illness, or indeed seeking help for oneself, was considered shameful and embarrassing in between 11 and 67% of adolescent respondents [36, 44, 47, 63, 71].

Lack of confidence in professional treatment and fear of being stigmatised by others were statistically significant factors affecting help-seeking in hypothetical scenarios [76] supported by descriptive qualitative and quantitative evidence that despite the perceived usefulness in theory, adolescents felt challenged to use professionals because of stigma [54, 71]. Compared to those without, willingness to seek help was statistically significantly lower in adolescents with pre-existing mental health problems [36]. Self-stigma was determined the strongest predictor of willingness to seek help in exploratory models incorporating literacy, mental health status and help-seeking attitudes [48]. Path analysis determined self-stigma mediates the relationship between illness severity and help-seeking attempts [78]. Contradictory evidence was found in one study that personal stigma was higher among those with no current or past mental health problems [61]. Observing others who seek help being themselves stigmatised, and discriminating against professionals from whom they sought help, emerged consistently from qualitative accounts [54, 65, 68]. Interestingly, stigma was rarely examined in relation to actual or intended help-seeking and many of the studies analysed quantitative data from convenience samples of CYP.

Poor recognition is a significant barrier to help-seeking [76] and conversely, greater recognition of mental health problems is significantly correlated with increasing likelihood of help-seeking [53]. Comparing stigma levels between CYP in two or more locations was widely investigated demonstrating that stigma levels can vary between countries and even between regions of the same country [37–40, 61, 76, 79]. Evidence of both strong and weak quality contributed to this finding. Interestingly, there was some consensus that females had more positive attitudes to help-seeking [72, 73, 80, 81] and held less stigmatising attitudes than males [37, 47, 79] among mixed quality sources. Regarding depression, studies that did report this outcome, had mixed findings [54, 61] while other studies provided weaker evidence of no such gender differences [34, 38].

#### Knowledge of how to access mental health information

Minimal data were obtained from this review regarding CYP knowledge about how to access mental health information [33, 38, 40, 51]. Survey data highlighted the importance of family and friends as a specific source of information for a subset of Chinese CYP sampled in two separate studies [38, 40] with qualitative data from 19 Ugandan adolescents adding that experiencing mental health problems, either their own or witnessing this through family and friends, significantly enhanced their understanding [33]. Chinese adolescents accessed mental health information through TV/movies, newspaper, magazines, and school [38, 40] and Vietnamese high-school students highlighted the growing importance of technologies including the internet and helplines as a key source of mental health information [51]. There was variation across different sites in how different sources of information were prioritised [38, 40] but understanding of how CYP accessed mental health information from other LMICs was largely absent.

## Discussion

This review analysed and synthesised empirical evidence regarding knowledge, beliefs and attitudes towards mental illness among CYP in LMICs drawing on Jorm’s framework for MHL. We found that knowledge of mental illnesses, treatments and help-seeking among populations in included studies was generally poor and that recognition of specific mental illnesses varied considerably in the studies that measured this outcome using vignette-based methods. Measures of general MHL, which delineate between assessments of individual knowledge separate from their beliefs [82], revealed a more consistent pattern of poor knowledge and low levels of awareness of mental health problems among CYP. We found evidence of pervasive stigma with high proportions of CYP endorsing statements signifying stigmatising attitudes towards those with mental illnesses. We also found some evidence of personal stigma and self-stigma which was linked with help-seeking propensity in a smaller number of studies. In terms of seeking out appropriate and effective help from formal sources, we found that seeking help from healthcare professionals was often not prioritised, however, knowledge of how to access formal help was limited and understudied. Instead, CYP cited the value of traditional healers as a mode of treatment and beneficial self-help practices for illness prevention supports the notion that social networks are an important source of mental health assistance in LMICs.

Methodological weaknesses were evident in included studies and the quality across studies varied significantly. Among quantitative analyses, fewer cross-sectional studies satisfactorily demonstrated obtaining a representative sample to evaluate the generalisability of their findings and many studies utilised measures of MHL, or some attribute, which did not have demonstrable cultural validity in the settings where they were administered. Similarly, the studies included using vignette-based approaches did not allow for direct comparison as there is no capacity to sum MHL levels or contrast between MHL domains [83]. Measures of general MHL, which incorporate knowledge and beliefs about mental illness terminology, causation, etiology, prognosis and course of illness are believed to overcome the limitations of vignette-based methodologies and more comprehensively address the MHL construct [82, 84]. However, these types of measures are also limited due to heterogeneity across measures, failure to incorporate validated constructs of knowledge, help-seeking and stigma [14] and a lack of specificity which arises from grouping a wide range of mental health problems together [85]. Importantly, these tools fail to reflect wide variability in cultural explanations for mental illness which we found in this review and establishing reliable and valid measures of MHL is important for future evaluations of MHL in different settings and contexts. There were additional conceptual limitations as most recognition studies focus on individual diagnosis, i.e. depression, rather than evaluating the range of possible mental illnesses or concerns that could be raised by CYP [18, 84, 85] or acknowledging that mental health conditions and outcomes are not universally valid across different cultures [86].

What can be clearly seen from the synthesised evidence is the dominance of stigmatised beliefs among CYP perspectives. Stigma emerged as a substantive concept underlying knowledge and beliefs across several domains of MHL incorporating negative beliefs about mental illnesses, risk factors and causation, self-help and professional help-seeking. Strong evidence emerged that CYP misconceptions that mental illnesses are caused by spiritual and religious forces, witchcraft, wizardry or sorcery as a result of perceived wrongdoing are pervasive and linked with beliefs that experiencing a mental illness is shameful and embarrassing [41, 51]. Cultural factors were found to influence beliefs and attitudes towards people with mental health problems and affect willingness to seek help [61, 71] and we also found evidence of geographical variation when high- and low-income settings were compared but also when regions within the same country were contrasted [37, 40]. Wider literature depicts theories of stigmatisation that highlight how stereotypical beliefs are linked with prejudice and discrimination [87] and clear evidence exists of the relationship between stigma, treatment avoidance and delay in seeking help [20, 23]. Evidence from multiple systematic reviews shows that attitudes and perceptions are particularly important in determining whether adolescents propose to seek professional help for a mental health problem [15, 16, 88]. Stigma, being ashamed and fearing negative evaluations by others are significant barriers to help-seeking [15, 16, 89]. However, research of this nature is primarily conducted in high-income settings supporting earlier assertions that the field is dominated by research from Western, developed nations [14]. There is a dearth of evidence from CYP in LMICs that help in understanding the role of stigma in help-seeking and pathways to receiving effective care [15, 16, 90]. The present review supports some existing findings that stigma influences help-seeking, but none of the included studies measured actual help-seeking. There was some evidence that both self-stigma and public stigma directly influence willingness to seek help [48, 78], but overall the relationship between stigma and help-seeking was under-researched.

As described above, CYP tended towards identifying spiritual and religious leaders and traditional healers as sources of professional support for mental health problems. We found some evidence that CYP attributed greater value to these sources of support than healthcare professionals who were less frequently prioritised. In many cultures, traditional and lay healers are recognised as legitimate sources of help and assume a substantial role in mental health care delivery [85, 86]. Knowledge of varied sources of professional help was mixed and limited to a small number of studies. A single source of evidence found that previous positive experiences with mental health professionals was a prominent facilitator of help-seeking among young people consistent with reviewed evidence [89] although this finding needs replicating as it may have particular salience for the development of social contact interventions to reduce stigmatisation of professional mental health services and increase help-seeking for effective treatment [88, 91].

### Recommendations for future research

Several findings regarding the relationships between stigma, help-seeking and knowledge were restricted to individual studies in this review indicating the need to more fully evaluate attributes of MHL and evaluate interrelationships to more fully understand the interplay between knowledge, beliefs and attitudes across varied cultural settings. Campaigns focusing on improving mental health knowledge have consistently demonstrated that public awareness can be improved while changing negative attitudes about mental illness proves a more challenging target [92]. Although we found evidence of pervasive stigma, few studies meaningfully evaluated and captured the varied forms of stigma fully despite the need to appreciate the role of different types on individual help-seeking. Evidence shows that specific stigma types exert greater influence on active help-seeking than others [23] which is important for targeted stigma reduction campaigns aimed at promoting help-seeking. Comparable to the global literature, conceptualisation and measurement of facets of MHL theory concerning knowledge and beliefs about self-help and how to access mental health information for wellbeing in LMICs were less complete than other facets [14, 83]. Future research should focus on understanding the complexities of help-seeking, particularly active help-seeking exploring varied sources (informal, formal, self-help) and their relationship to the types of problems for which help is being sought. Additionally, a review of the current strategies used for stigma reduction, delivered and tested for their impact on CYP in LMICs, demonstrates that evidence-based interventions for these groups are disappointingly scarce [91]. Regarding sources of help, the value of collaborating with traditional healers needs greater exploration in younger populations in addition to appreciating the value of social networks to identify community platforms for educational interventions [93]. Community interventions are likely to have even greater relevance in low-resource settings [91, 94] whilst continuing to recognise the need to develop interventions across socio-ecological levels is key, particularly for child and adolescent populations [91].

### Strengths and weaknesses

MHL theory has considerable utility as a structure for understanding and explicating factors that influence individuals help-seeking in relation to their mental health [82]; however, it has been described as an approximation of a theory [18]. Consensus has yet to be achieved regarding the dimensional structure of each MHL attribute [18, 85] and there is conceptual confusion regarding delineating the MHL construct and potential outcomes, appreciating what MHL is and what it is not, which have hampered coordinated research efforts [14]. Evidence indicates there are no tools that adequately evaluate all attributes and some measure additional constructs, such as knowledge and beliefs about course of illness, treatment outcome and recovery not included in original descriptions of the construct [82]. An important argument is that current theoretical approaches, whether measuring one or several MHL domains, fail to adequately and logically theorise the processes and mechanisms of action that link attitudes, intentions and behaviour [85]. We found that this lacking among the studies in the present review and similarly, we found no study that evaluated all facets of MHL which is important for developing research going forward. Another recent review, which focuses on MHL conceptualisation for adolescents, highlights similar constraints in that researchers purporting to examine MHL explored various adaptations and interpretations of the construct [14].

Another issue with measures denoting the assessment of knowledge is that they appear to tap into how individual respondents perceive illnesses to emerge and how they assess the usefulness of professional and lay forms of help [82]. Gauging whether respondents have the capacity including the necessary knowledge and skills to appraise the need for different types of help and prioritise help-seeking routes and strategies is often overlooked among assessments of MHL [82, 85] and is particularly pertinent to the inclusion of knowledge about self-management strategies [12]. Jorm’s theory [12] promotes self-management for minor problems but within conceptualisations of help-seeking which encompass illness behaviour, self-help can be understood to refer to how people monitor their own health, define and interpret their symptoms and take preventive or remedial action which may also incorporate using healthcare systems [85]. Essentially, the range of possible ways that people can triage their own mental health concerns and address these is not encompassed within this existing theory and should be considered for future conceptualisations of MHL constructs.

This review is strengthened by the use of a comprehensive search strategy, rigorous approach to screening, extraction and synthesis and guiding analysis with existing theory. The MMAT quality appraisal tool was employed to assess the quality of included studies highlighting significant variation in quality of published research in this area which can be considered a limitation. Developing culturally appropriate measures of MHL is key to enhancing the methodological rigour of research in LMICs while remaining sensitive to cultural variation in different settings. There is a potential for narrative synthesis to de-contextualise findings and we aimed to mitigate against this using a robust process for analysis underpinning synthesised data with in-depth qualitative findings where possible. Lastly, we identified that most studies in this synthesis did not include participants under the age of 10 despite including younger groups in our selection criteria. This may be due to the developmental aetiology of common mental illnesses where age of onset is concentrated in the middle and later stages of adolescence [5]. However, there is a need for research globally to understand and enhance mental health literacy for greater detection of childhood disorders [95] in addition to developing interventions for CYP.

## Conclusion

This review highlights important issues regarding MHL for CYP in LMICs including low levels of recognition and knowledge about mental health problems and illnesses, pervasive levels of stigma and low confidence in professional healthcare services even when considered a valid treatment option. CYP cited the value of traditional healers and social networks for seeking help. There were several areas that were under-researched including the link between specific stigma types and active help-seeking and research is needed to understand more fully the interplay between knowledge, beliefs and attitudes across varied cultural settings. Greater exploration of social networks and the value of collaboration with traditional healers is consistent with promising, yet understudied, areas of community-based MHL intervention combining education and social contact.

## Data statement

The authors confirm that the data supporting the findings of this study are available within the article and its supplementary materials.

### Supplementary Information

Below is the link to the electronic supplementary material.

Supplementary file1 (DOCX 15 kb)

Supplementary file2 (DOCX 14 kb)

## Footnotes

1 DAC recipient countries are those eligible to receive official development assistance (ODA) based on gross national income (GNI) per capita as published by the World Bank.

## Declarations

### Reflexivity statement

LR is a Senior Lecturer in Mental Health Nursing, HB a Senior Lecturer in Health Services Research, PB a Professor of Health Services Research, KL a Professor in Mental Health, IJ a Psychology Researcher, VB a Research Fellow and RP a Senior Research Fellow. The starting point for the research was one informed by the value of mental health literacy for children and adolescents in low- and middle-income countries recognising that deficit approaches need to be evaluated and considered for their usefulness in these settings. We also took a position that positive views of mental health contribute to how mental health perceptions are constructed and may influence access and treatment. Analysis and synthesis were conducted primarily by LR, HB and RP with significant experience of evidence synthesis and both quantitative and qualitative research.
