# Supplementary material for: Mental health literacy in children and adolescents in low- and middle-income countries: a mixed studies systematic review and narrative synthesis
Source: Eur Child Adolesc Psychiatry. 2022 May 15;33(4):961–85. doi: 10.1007/s00787-022-01997-6 (PMC11032284; doi:10.1007/s00787-022-01997-6)
Supplement: Supplementary file 1 — Supplementary file1 (DOCX 15 kb) [file 787_2022_1997_MOESM1_ESM.docx]

Additional File 1: Inclusion and Exclusion Criteria

| Category | Inclusion | Exclusion |
| --- | --- | --- |
| Population of Interest | - Views, attitudes and perceptions of under 18-year old's towards mental health, emotional well-being and treatment seeking for mental health problems (where children and young people are employed the mean age of the sample will be less than 18 years old). - Data collected within a low/middle income country (as defined by OECD’s DAC list for 2018 to 2020). | - Data obtained representing the views of CYP, parents, teachers or other professional where individual CYP data cannot be extracted. - Data collected in high income countries employing ethnic minorities originating from low/middle income countries (as defined by OECD's DAC list for 2018 to 2020). - Studies where the primary research question is about developmental disorders. |
| Study Types and Designs | - Primary data from observational studies, cross-sectional data, surveys, other non-experimental quantitative research, and qualitative and mixed methods studies were included. | - Not primary data - Data from reviews |
| Health Outcomes and Outputs of Interest | - Perceptions and views to include knowledge, attitudes (e.g. stigma), beliefs and mental health literacy adopting Jorm’s concept of mental health literacy encompassing - the ability to recognise specific disorders or different types of psychological distress - knowledge and beliefs about risk factors and causes - knowledge and beliefs about self-help interventions - knowledge and beliefs about professional help available - actors and attitudes which facilitate recognition and appropriate help-seeking - knowledge of how to seek mental health information | - Studies that do not measure the outcome of interest |
| Publication Dates | All publication dates |  |
| Publication Languages | All languages |  |
