# Supplementary material for: Mental health literacy in children and adolescents in low- and middle-income countries: a mixed studies systematic review and narrative synthesis
Source: Eur Child Adolesc Psychiatry. 2022 May 15;33(4):961–85. doi: 10.1007/s00787-022-01997-6 (PMC11032284; doi:10.1007/s00787-022-01997-6)
Supplement: Supplementary file 2 — Supplementary file2 (DOCX 14 kb) [file 787_2022_1997_MOESM2_ESM.docx]

Additional File 2: Example Search Strategy

1. "Child$" OR "Adolescen$" OR "Teen$" OR "Juvenile$" OR "Boy$" OR "Girl$" OR "School child$" OR "Youth$" OR "Young person$" OR "Young people" OR "Youngster$" OR "paedriatric" OR "pediatric" OR “paediatric” OR “School-age” AND

2. (MH "Perception") OR "Perception" OR "Perspective" OR (MH "Public Opinion") OR "Public opinion" OR "Community Attitudes" OR "Child attitudes" OR "Adult Attitudes" OR (MH "Parental Attitudes") OR (MH "Student Attitudes") OR "Adolescent Attitudes" OR "Client Attitudes" OR "Teacher Attitudes" OR (MH "Attitude to Mental Illness") OR (MH "Attitude") OR "Attitude" OR "View" OR "belief" OR "Believe" OR "Interpretation" OR "Mental health literacy” AND

3. "Mental disorder" OR "Mental illness" OR "Mental distress" OR (MH "Mental Health") OR "mental health" OR "emotional well-being" OR "Mental well-being" OR (MH "Psychological Well-Being") OR "psychological well-being" OR "serious mental illness" OR "severe mental illness" OR "common mental health problems" OR (MH "Mental Disorders") AND

4. (MH "Low and Middle Income Countries") OR "Low-income countries" OR "Middle-income countries" OR "Low Income Countries" OR "Middle income countries" OR "Low & middle Income Countries" OR "Low and middle-income countries" OR "Low- and middle-income countries" OR "Low-income and middle-income countries" OR "Lower income level" OR "Middle income level" OR "LAMIC" OR "Afghanistan" OR "Albania" OR "Algeria" OR "Angola" OR "Antigua and Barbuda" OR "Argentina" OR "Argentinian" OR "Armenia" OR "Azerbaijan" OR "Bangladesh" OR "Belarus" OR "Belize" OR "Benin" OR "Bhutan" OR "Bolivia" OR "Bosnia" OR "Botswana" OR "Brazil" OR "Burkina Faso" OR "Burundi" OR "Cabo Verde" OR "Cambodia" OR "Cameroon" OR "Central African Republic" OR "Chad" OR "China" OR "Chinese" OR "Colombia" OR "Comoros" OR "Comorian" OR "Congo" OR "Cook Islands" OR "Costa Rica" OR "Côte d'Ivoire" OR "Cote d'Ivoire" OR "Cuba" OR "Djibouti" OR "Dominican Republic" OR "Ecuador" of "Eqypt" OR "El Salvador" OR "Equatorial Guinea" OR "Eritrea" OR "Ethiopia" OR "Fiji" OR "Gabon" OR "Gambia" OR "Georgia" OR "Ghana" OR "Grenada" OR "Grenadian" OR "Guatemala" OR "Guinea" OR "Guinea-Bissau" OR "Guyan" OR "Haiti" OR "Honduras" OR "Honduran" OR "India" OR "Indonesia" OR "Iran" OR "Iraq" OR "Jamaica" OR "Jordan" OR "Kazakhstan" OR "Kazakh" OR "Kenya" OR "Kiribati" OR "North Korea" OR "North Korean" OR "Kosovo" OR "Kyrgyzstan" OR "Kyrgyz" OR "Lao" OR "Loas" OR "Lebanon" OR "Lebanese" OR " Lesotho" OR "Liberia" OR " Libya" OR "Macedonia" OR "Madagascar" OR "Madagascan" OR "Malawi" OR "Malaysia" OR "Maldives" OR "Mali" OR "Marshall Islands" OR "Mauritania" OR "Mauritius" OR "Mexico" OR "Mexican" OR "Micronesia" OR "Moldova" OR "Mongolia" OR "Montenegro" OR "Montenegrin" OR "Montserrat" OR "Morocco" OR "Moroccan" OR "Mozambique" OR "Myanmar" OR "Namibia" OR "Nauru" OR "Nepal" OR "Nicaragua" OR "Niger" OR "Nigeria" OR "Niue" OR "Pakistan" OR "Palau" OR "Panama" OR "Papua New Guinea" OR "Paraguay" OR "Peru" OR "Philippine" OR "Filipino" OR "Rwanda" OR "Saint Helena" OR "Samoa" OR "São Tomé and Príncipe" OR "Sao Tome and Principe" OR "Senegal" OR "Serbia" OR "Sierra Leone" OR "Solomon Islands" OR "Somalia" OR "South Africa" OR "South Sudan" OR "Sri Lanka" OR "Saint Lucia" OR "Saint Vincent" OR "St Vincent" OR "Grenadines" OR "Sudan" OR "Suriname" OR "Swaziland" OR "Swazi" OR "Syria" OR "Tajikistan" OR "Tanzania" OR "Thailand" OR "Thai" OR "Timor-Leste" OR "Togo" OR "Tokelau" OR "Tonga" OR "Tunisia" OR "Turkmenistan" OR "Turkey" OR "Turkish" OR "Tuvalu" OR "Uganda" OR "Ukraine" OR "Ukrainian" OR "Uzbekistan" OR "Vanuatu" OR "Venezuela" OR "Vietnam" OR "Wallis and Futuna" OR "West Bank and Gaza Strip" OR "Yemen" OR "Zambia" OR "Zimbabwe" OR "Hong Kong"
